# Edge effects in fire‐prone landscapes: Ecological importance and implications for fauna

**DOI:** 10.1002/ece3.4076

**Published:** 2018-05-08

**Authors:** Kate Parkins, Alan York, Julian Di Stefano

**Affiliations:** ^1^ School of Ecosystem and Forest Sciences University of Melbourne Creswick Vic. Australia

**Keywords:** animal movement, biodiversity, disturbance, faunal conservation, permeability, prescribed fire, wildfire

## Abstract

Edges are ecologically important environmental features and have been well researched in agricultural and urban landscapes. However, little work has been conducted in flammable ecosystems where spatially and temporally dynamic fire edges are expected to influence important processes such as recolonization of burnt areas and landscape connectivity. We review the literature on fire, fauna, and edge effects to summarize current knowledge of faunal responses to fire edges and identify knowledge gaps. We then develop a conceptual model to predict faunal responses to fire edges and present an agenda for future research. Faunal abundance at fire edges changes over time, but patterns depend on species traits and resource availability. Responses are also influenced by edge architecture (e.g., size and shape), site and landscape context, and spatial scale. However, data are limited and the influence of fire edges on both local abundance and regional distributions of fauna is largely unknown. In our conceptual model, biophysical properties interact with the fire regime (e.g., patchiness, frequency) to influence edge architecture. Edge architecture and species traits influence edge permeability, which is linked to important processes such as movement, resource selection, and species interactions. Predicting the effect of fire edges on fauna is challenging, but important for biodiversity conservation in flammable landscapes. Our conceptual model combines several drivers of faunal fire responses (biophysical properties, regime attributes, species traits) and will therefore lead to improved predictions. Future research is needed to understand fire as an agent of edge creation; the spatio‐temporal flux of fire edges across landscapes; and the effect of fire edges on faunal movement, resource selection, and biotic interactions. To aid the incorporation of new data into our predictive framework, our model has been designed as a Bayesian Network, a statistical tool capable of analyzing complex environmental relationships, dealing with data gaps, and generating testable hypotheses.

## 1 INTRODUCTION

Edges, barriers, boundaries, or ecotones are ubiquitous environmental phenomena, occurring in a wide range of ecosystems and across multiple spatial scales (Cadenasso, Pickett, Weathers, Bell et al., 2003). Edges can occur both within and between land cover types and refer to the interface or transition zone between areas of differing structural characteristics. Edges can be dynamic or static and frequently exhibit greater heterogeneity than adjacent areas (Peters et al., 2006; Yarrow & Salthe, 2008). Edges are ecologically important because they influence a wide range of patterns and processes (Ries, Fletcher, Battin, & Sisk, 2004). The resulting ecological changes at edges are collectively known as edge effects.

Edge effects result from both abiotic (e.g., radiation, moisture, temperature) and biotic (e.g., species interactions) subprocesses that interact to generate environments with different structural attributes and species assemblages compared to other parts of the landscape (Craig, Stokes, Hardy, & Hobbs, 2015; Murcia, 1995). A key aspect of edges is their capacity to influence the flow of energy and materials. They have been described as ecological analogues to cellular membranes (Harper et al., 2005), being semi‐permeable boundaries that allow certain materials or organisms to flow freely while restricting or prohibiting the movement of others (Laurance, Didham, & Power, 2001). Understanding this aspect of edges is critical as movement processes influence the fate of individuals and the structure and function of ecosystems (Fahrig, 2007; Nathan et al., 2008).

Edge effects have been extensively researched in some contexts, particularly in highly modified agricultural and urban landscapes, and have been the subject of several reviews (see Cadenasso, Pickett, Weathers, Bell et al., 2003; Harper et al., 2005; Murcia, 1995; Ries et al., 2004). While significant progress has been made in the study of edges, several aspects remain poorly understood. For example, fire is an agent of edge creation and a globally important driver of biome distribution and community composition (Bond & Keeley, 2005; Pastro, Dickman, & Letnic, 2011), yet little is known about how fire edges affect ecological processes in flammable ecosystems. Here, we outline this knowledge gap, focusing on the potential influence of fire edges on animals. We then describe a conceptual model for predicting the edge response of animals in flammable landscapes and present an agenda for future research.

**Figure 1:**
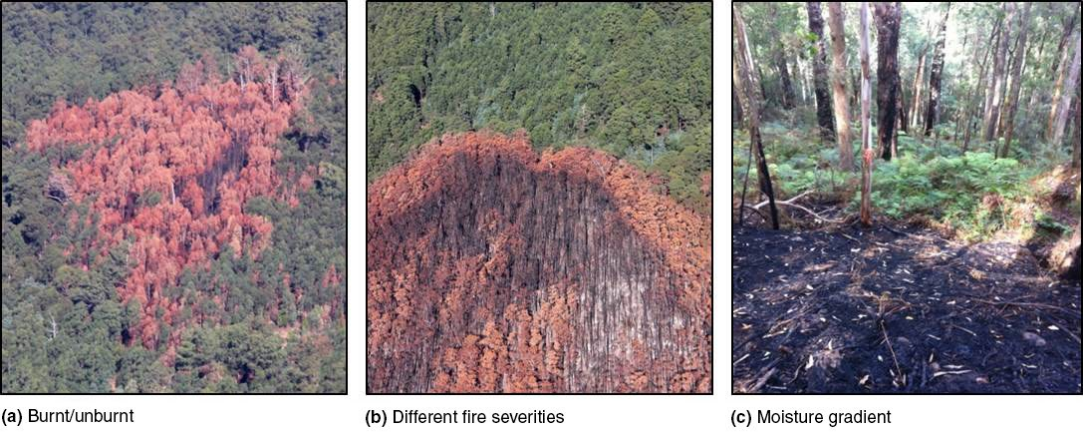
Different types of fire edges. (a) Edges between burnt/unburnt, (b) edges between differing fire severities (i.e., unburnt, moderate burn and high severity burn), (c) edges at moisture gradients where the fuel becomes less flammable. Note‐ in image (c) the edge‐zone for this fire can be clearly seen; however, there are several trees within the unburnt section that retain fire scars from a previous fire. These fire scars illustrate the temporal and spatial variability in edge locations (Images: a and b—DELWP, 2015; c Parkins, 2015)

We define a fire edge as an interface or transition zone generated by fire, resulting in a boundary between areas of differing structural characteristics. This may refer to edges between burnt and unburnt vegetation, between different burn severities, or adjacent areas burnt at different times or frequencies. Several types of fire edges are shown in Figure 1.

## 2 FIRE AS AN AGENT OF EDGE CREATION

Earth is intrinsically flammable (Bowman et al., 2009), with wildfires predicted to increase in extent and severity as a result of climate change (Flannigan, Krawchuk, de Groot, Wotton, & Gowman, 2009). In response to this, prescribed fire is increasingly being used as a management tool globally (Fernandes et al., 2013; Penman et al., 2011; Stephens et al., 2012). Given this likely increase in fire activity a better understanding of fire edges with respect to their ecological function and implications for animals is important. To date, no studies have quantified the impact of fire on architectural properties of edges such as shape and contrast, or how the characteristics of fire edges change in space and time.

Edges are created by fire through the consumption of fuel and the factors that cause fires to extinguish. In forested landscapes, there are many factors that interact to influence fire behavior and spread (Bradstock, Hammill, Collins, & Price, 2010) and determine the location and architecture of fire edges. Explicit consideration of the factors that cause fires to extinguish will be central to understanding the ecological dynamics occurring at fire edges, as different methods of extinguishment are likely to result in different edge characteristics (e.g., size, shape, contrast), influencing species edge response.

### 2.1 Fuel continuity

Variability in fuel structure and distribution is common in natural systems, and continuity of surface or near‐surface fuels is important for the spread of fire in most landscapes (Catchpole, 2002). Fires often extinguish in areas where the continuity of plant material is not sufficient to sustain burning, creating burnt/unburnt edges. Fuel discontinuities include areas of topographical change (i.e., cliff lines, rocky outcrops) often characterized by a sharp reduction in the amount of available fuel. They may also be created through the construction of fuel breaks or roads.

### 2.2 Fuel flammability

Gradients of moisture availability govern both the accumulation of fuel and its ability to burn (Bradstock, 2010). Topographic locations such as gullies, shaded aspects, or riparian zones are commonly more productive than other drier parts of a landscape, yet these areas often remain unburnt (or burnt at lower intensities). This is commonly due to higher fuel moisture, and/or because they support intrinsically less flammable vegetation, facilitating edge creation nearby. For example, patches of Chenopod Mallee which are less flammable than surrounding Triodia Mallee in southeastern Australia (Haslem et al., 2011), or patches of evergreen (Afromontane) forest surrounded by highly flammable fynbos shrubland in South Africa (Van Wilgen, Higgins, & Bellstedt, 1990).

### 2.3 Weather

Severe ambient weather conditions (i.e., strong winds, high temperatures, low humidity) contribute to the ease of ignition, rate of spread, pattern of fire intensity, and the location and architecture of fire edges. The area burnt at differing severities (or remaining unburnt) will be strongly influenced by these factors (Bradstock, 2010). For example, highly flammable areas may remain unburnt due to a sudden wind change redirecting the fire‐front.

### 2.4 Management actions

Fire edges can also result from human‐generated discontinuities in fuel caused by prescribed burning, construction of fuel breaks or roads, or from suppression activities during fire. Edges at fuel discontinuities (i.e., fuel breaks) and those resulting from suppression activities are likely to be higher contrast than edges resulting from natural processes, and more similar to edges in agricultural and urban landscapes.

## 3 EDGE EFFECTS IN FIRE‐PRONE LANDSCAPES

Fire‐generated edge effects are likely to differ from other edge types in several ways. They are temporally dynamic, spatially complex and are characterized by the strength of the interaction between components of the disturbance regime and other biophysical factors. While edge effects and faunal fire responses have been well studied independently, how animals respond to fire edges remains poorly understood. Here, we review the current state of knowledge and highlight several aspects of existing edge literature and fire research generally relevant to understanding interactions between animals, fire, and edges in flammable landscapes.

We conducted a literature search for papers that specifically investigated faunal responses to fire edges. We focused our search on studies that defined an edge zone or included distance from edge in the analysis. We searched for papers in the Web of Science, using the following search criteria: TOPIC: (“fire edge effects” OR “fire edge” OR “burn edge” OR edge) AND (fire OR wildfire) AND (fauna OR animals) and sorted the results by relevance, not excluding any years. We also included relevant papers cited in reference lists. Here, we summarize the key findings.

### 3.1 Fire edges and edge effects are temporally dynamic

Edges in modified systems (e.g., between pasture and forest) are often maintained at a relatively stable state, but this is not the case for fire edges. Edges resulting from fire are in a constant state of flux due to (1) postfire regeneration; and (2) the occurrence of new fires. Fire edges are ephemeral, dynamic parts of fire‐affected landscapes, where an edge zone may be impermeable to some species immediately after fire, but highly permeable at a certain point in time post‐fire. However, it is not the time per se that is important in determining faunal succession (Monamy & Fox, 2000), but the pattern of resource regeneration and reaccumulation.

Some animals, including birds, small mammals, and reptiles, avoid or minimize time spent at hard edges (high contrast) in modified landscapes (Goosem, 2001; Laurance, Goosem, & Laurance, 2009; Lehtinen, Ramanamanjato, & Raveloarison, 2003; Wilson, Stirnemann, Shaikh, & Scantlebury, 2010). Although data are lacking, similar responses may occur at fire edges immediately post‐fire, where the contrast between the burnt and unburnt side of the edge is often high. Unlike edges in modified landscapes, we would expect these effects to reduce over time as vegetation regenerates and the fire edge softens. Some small mammal species have been shown to avoid fire edges for 4–5 years postfire, until shrub cover and seed production reached sufficient levels to sustain viable populations in these areas (Borchert & Borchert, 2013). In contrast, reduced understory cover post‐fire can enable predators to hunt more effectively (Conner, Castleberry, & Derrick, 2011; Hradsky, Mildwaters, Ritchie, Christie, & Di Stefano, 2017; Leahy et al., 2016), resulting in the increased prevalence of some predators at fire edges briefly post‐fire, while the contrast between burnt and unburnt remains high.

The temporal extent of a fire edge effect will also be influenced by species‐specific factors. Early successional post‐fire habitats are characterized by vigorous growth of understorey vegetation and are often dominated by ground‐foraging herbaceous mammal species. As the cover of woody plants increases over time, granivorous species and those that feed on invertebrates have been shown to move into the regenerating landscape (Torre & Díaz, 2004). A study in chaparral shrublands (USA) at a high‐contrast burnt/unburnt edge along the perimeter of a wildfire found edge response differed between species with differing resource requirements. A habitat generalist (pinyon mouse, *Peromyscus maniculatus*) was found to occupy burnt edge and unburnt habitat in similar abundance within 1 year of a high‐intensity wildfire. In contrast, a late seral specialist (Californian mouse, *Peromyscus californicus*) was more prevalent in unburnt habitat, took 4–5 years to occupy the edge zone and was only detected in high abundance in burnt vegetation nine years after fire (Borchert & Borchert, 2013). Burnt chaparral produces an abundance of seeds immediately after fire and this is likely driving the high occupancy of the burnt site by granivorous species or those with generalist habitat and/or diet requirements. However, unburnt chaparral provides better protection from predators, especially early post‐fire. This asymmetry in food and habitat resources across burnt/unburnt edges creates conditions in which some species may increase their use of fire edges (Sitters et al., 2014), with access to abundant food on the burnt side and protection from predators on the unburnt side.

### 3.2 Fire edges and edge effects are spatially variable

Fire edges form at multiple spatial scales, occurring as external perimeters to a single‐fire event (external burn edge), within the boundary of a single‐fire event (internal burn edges), or as temporally overlapping edges between multiple fires. Fire events are often patchy, with different parts of the landscape being burnt at high, moderate or low severity, or remaining unburnt. Large‐scale, high‐severity fires can produce a spatially diverse mosaic of different burn intensities (Arthur, Catling, & Reid, 2012; Bradstock, 2008) and prescribed fire in relatively homogenous landscapes produce patchy outcomes (Penman, Kavanagh, Binns, & Melick, 2007). Many fire management strategies have been designed to deliberately increase variability through the use of dynamic fire mosaics across space and time (Bradstock, Bedward, Gill, & Cohn, 2005; Parr & Andersen, 2006).

Edge effects resulting from fires are expected to be shorter lived than edges in highly modified landscapes due to the dynamic nature of post‐fire regeneration. However, repeated, unpredictable disturbances like fire can produce a mosaic of patches at different successional stages, resulting in multiple, overlapping edges which may substantially influence species distributions and community structure (Wiens, 1976).

In modified landscapes, the magnitude and extent of edge effects have been shown to increase at locations where several edges are present (Fletcher, 2005). In fire‐prone landscapes, multiple fires result in an intricate network of fire edges, each with a unique trajectory of temporal change. How an animal responds to a fire edge may be a function of the edge itself and/or the context of that edge within the broader landscape. For example, northern spotted owls (*Strix occidentalis caurina*) were found to be attracted to hard edges caused by severe fire and salvage logging when these were small patches within a larger area burnt at low severity (Comfort, Clark, Anthony, Bailey, & Betts, 2016). However, for this species, the influence of both hard and soft edges on habitat selection depended on spatial scale. With the exception of attraction to hard edges at a small spatial scale, northern spotted owls were generally attracted to soft edges and avoided hard edges (Comfort et al., 2016). Soft edges were characterized by an intact canopy and regenerating shrub layer, resulting in structurally complex forest promoting prey availability and facilitating hunting under a closed canopy. Hard edges were characterized by high severity burnt patches, some of which had been salvage logged, adjacent to forest burnt at low severity. These areas likely supported fewer prey and resulted in a suboptimal hunting environment.

Studies at edges in fragmented landscapes have shown species to be more strongly associated with local habitat resources rather than edge structure (Schultz, Franco, & Crone, 2012; Villasenor, Blanchard, Driscoll, Gibbons, & Lindenmayer, 2015), and this is likely to be similar for many species at fire edges. Small mammal abundance both close to and distant from a fire edge was found to be influenced by site‐specific factors such as the presence of riparian zones, topography, and shrub species composition (Diffendorfer, Fleming, Tremor, Spencer, & Beyers, 2012). It is therefore important to consider fire edges within the context of other edges in the surrounding landscape (Cochrane & Laurance, 2002).

### 3.3 Responses to fire edges are species‐specific

Species‐specific traits and resource requirements are major drivers of post‐fire recovery and recolonization and are likely to play a key role in how certain species respond to fire edges. Species with preferences for disturbed and/or open conditions have been shown to dominate recently burnt sites, whereas species with preference for older vegetation are more prevalent at unburnt sites (Diffendorfer et al., 2012).

Small species with limited mobility may find edges difficult to cross until the vegetal components vital to their survival regenerate to sufficient levels (Santos, Bros, & Mino, 2009). Fire‐induced edge effects can last for several years for species strongly associated with microhabitat structure. For example, burnt sites were characterized by reduced gastropod species richness for up to 4 years after fire (Santos et al., 2009) and reduced beetle community composition for up to 5 years (Elia, Lafortezza, Tarasco, & Sanesi, 2016). In contrast, larger, highly mobile animals such as the Canada lynx (*Lynx canadensis*) have been shown to preferentially select fire edges during the first year post‐fire because of the contrasting vegetation characteristics provided by the burnt/unburnt edge (Vanbianchi, Murphy, & Hodges, 2017). Similarly, Californian spotted owls (*Strix occidentalis occidentalis*) were found to have a higher probability of selecting fire edges than contiguous habitat, with survival and reproductive rates higher in areas containing edge habitat (Eyes, Roberts, & Johnson, 2017).

In situ survival and ex situ colonization may drive post‐fire recovery and edge response. Distance from fire edge had little or no effect on small mammal abundance in either chaparral shrub land or tall wet forest (Banks et al., 2011; Diffendorfer et al., 2012), nor did it affect species richness or abundance of several cockroach species in foothills forest (Arnold, Murphy, & Gibb, 2017). However, post‐fire recovery of some litter detritivore species was found to be limited by distance from burn edge, with this variable an important determinant of post‐fire assemblages up to 6 years after fire (Arnold et al., 2017). Species richness of birds in semi‐arid Mallee shrublands was also found to be higher at sites closer to unburnt vegetation and at sites containing unburnt patches, suggesting that colonization from ex situ populations was an important process for the recovery of avifauna post‐fire in this system (Watson et al., 2012). These studies suggest that in some cases surviving individuals are driving population recovery in burnt areas, while in others distance‐from‐edge, edge permeability, and subsequent recolonization from unburnt areas are important.

Responses to fire edges are likely to be species, not taxa specific. For example, bark‐probing woodpecker species such as black‐backed woodpeckers (*Picoides arcticus*) and hairy woodpeckers (*Picoides villosus*) are attracted to recently burned areas because of increases in wood‐boring beetles (Vierling, Lentile, & Nielsen‐Pincus, 2008), with higher reproductive success at edges than deep in burnt forest (Nappi & Drapeau, 2009). In contrast, other species of woodpeckers have been shown to preferentially nest further from the edge of burnt patches as a predator avoidance strategy (Vierling et al., 2008).

### 3.4 Responses to fire edges involve complex interactions

Edges created by fire never occur in isolation from other environmental patterns and processes. Accordingly, fire edges are inherently complex due to these interactions with other variables. Edge effects may be exacerbated, diminished, or masked by the interaction of other factors, making it difficult to determine if animals are responding independently to a fire edge effect or to other processes. Much of the existing fire edge data has been collected at pre‐existing edges (e.g., between forest and highly modified agricultural land) that were subsequently affected by fire (see Figueiredo & dos Santos Fernandez, 2004; Mendes‐Oliveira et al., 2012; Pires, Fernandez, de Freitas, & Feliciano, 2005). In these studies, fire‐induced edge effects were confounded by the presence of hard, anthropogenically modified edges, making it difficult to determine whether animals were responding to pre‐existing forest/farmland edges or burnt/unburnt edges. However, for some species, it may be this unique interaction between fire and other processes that enables them to utilize edge habitat. For example, the location of greater prairie‐chicken (*Tympanuchus cupido*) lek sites (areas for communal courtship display) was influenced by an interaction between patch edge and fire, with leks positioned near patch edges at recently burnt sites (Hovick et al., 2015). Furthermore, processes such as herbivory may interact with fire edge effects to influence the spatio‐temporal dynamics of fire edges. Herbivores are often attracted to the flush of vegetation growth post‐fire because of enhanced forage quality and increased productivity in burnt areas (Eby, Anderson, Mayemba, & Ritchie, 2014; Wilsey, 1996). However, post‐fire grazing may intensify or prolong fire‐induced edge effects, changing the nature of species’ interactions and influencing species’ responses to these edges.

## 4 PREDICTING EDGE EFFECTS IN FLAMMABLE LANDSCAPES

Current models predicting the response of fauna to edges are predominantly based on the distribution and quality of resources in adjoining habitats (see Cadenasso, Pickett, Weathers, & Jones, 2003; Ries et al., 2004). While these models have proven effective in predicting edge effects across a range of environments, they are unlikely to perform well in flammable ecosystems for two reasons.

Firstly, these models do not explicitly consider the process of edge creation and we argue that edge effects cannot be effectively understood in isolation from the processes that generate them. This is of particular importance for edges created by fire where temporal changes in edge architecture are expected to be influenced by fire regime attributes and both static (e.g., topography) and dynamic (e.g., climate) biophysical properties. Fire affects both the horizontal and vertical composition of vegetation through flame height and radiant heat, resulting in three‐dimensional edge effects (depth, length, and height). Understanding how a disturbance interacts with biophysical factors to influence the physical characteristics of an edge will be essential for predicting edge permeability, species edge response and how edge effects might change in time and space.

Secondly, while current predictive models suggest that edge response will differ between mobile and sessile organisms (Ries et al., 2004), we argue that consideration of traits that go beyond mobility will be important predictors of species edge response in flammable systems. Faunal traits influence species susceptibility to environmental change and predispose some species to decline at a greater rate than others in the face of adverse environmental shifts (Pimm, Jones, & Diamond, 1988; Webb, Hoeting, Ames, Pyne, & LeRoy Poff, 2010). Quantifying interactions between traits and extrinsic factors can improve the capacity to predict species responses to threatening processes (Murray, Rosauer, McCallum, & Skerratt, 2011). Understanding how traits interact with edge characteristics in a changing landscape, and how this influences movement, biotic interactions and access to resources for different species may enhance the predictive capacity of edge effects models.

## 5 CONCEPTUAL MODEL

**Figure 2:**
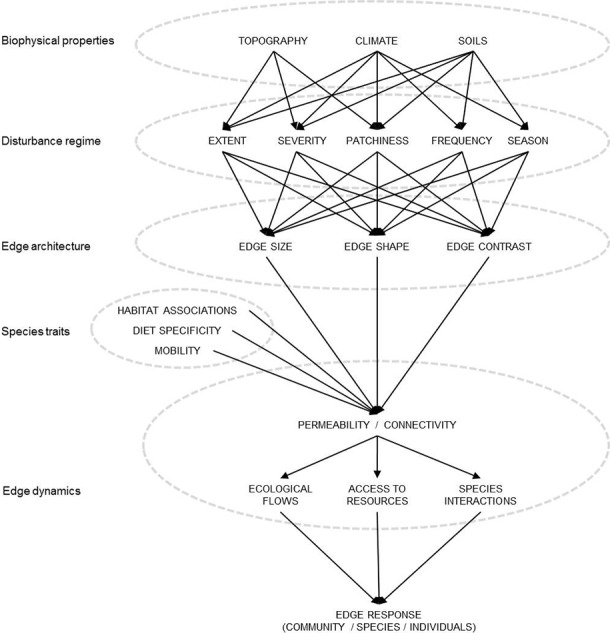
A conceptual model of the factors driving edge effects in fire‐prone landscapes. The model considers the origin of edge creation, including biophysical factors and elements of the disturbance regime. Interactions between these factors influence edge architecture (edge size, shape, and contrast), which influences edge dynamics (such as site permeability and landscape connectivity). Species traits such as the strength of habitat associations, diet specificity, and mobility will also contribute to the dynamics occurring at fire edges. The unique interaction of all of these variables will influence how individual animals, species, or communities respond to fire edges. The direction of arrows indicates the direction of influence

In response to the issues identified above, we have developed a conceptual model (Figure 2) for predicting how fire is likely to shape the physical properties of an edge and influence species edge responses. While this model was designed to predict the responses of fauna to fire edges, it could also be applied to other disturbance contexts, as components of the disturbance regime can be modified to suit any edge creation process that results in a changed landscape (i.e., naturally or anthropogenically occurring). For the purposes of this paper, we discuss the components of the model and their interactions using fire as the disturbance process.

### 5.1 Biophysical properties

In fire‐prone landscapes, the location of unburnt patches often occurs in a non random manner, and this is largely due to variations in topography, climate and soils, and their influence on fire behavior. Topographic locations with higher fuel moisture may experience lower fire severity and intensity and have a lower probability of burning than adjacent drier topographies (Bradstock, 2010; Collins, Bradstock, Tasker, & Whelan, 2012; Penman et al., 2007). Topography is therefore an important biophysical feature influencing where fire edges occur.

At large spatial scales, long‐term climatic fluctuations are correlated with the probability of fire ignition and spread, whereas at a smaller spatial scales local weather conditions (particularly temperature and wind speed) can influence fire behavior (Alexander, Seavy, Ralph, & Hogoboom, 2006), and therefore the position and physical characteristics of fire edges. Post‐fire rainfall contributes to the rate of vegetation recovery which will determine temporal changes in edge contrast. Furthermore, variations in soil moisture and nutrient levels contribute to heterogeneous patterns of vegetation, which in turn influence edge location and architecture.

### 5.2 Disturbance regime

Fire regimes incorporate the effects of discrete fire events with the cumulative effect of multiple fires over time and are characterized by spatially variable patterns in fire type, severity, spatial extent, patchiness, frequency and seasonality (Gill, 1975). Fire regimes generate a spatially and temporally shifting pattern of patches (Parr & Andersen, 2006) and their effect on animals is usually considered in this context (Griffiths, Garnett, & Brook, 2015; Kelly, Bennett, Clarke, & McCarthy, 2015; Taylor et al., 2012). However, patches have edges, and edge characteristics are likely to influence both fine‐scale movements and the broader distribution of many species.

Fire extent refers to the overall size of a fire and is predominately determined by biophysical properties. Extent is correlated with perimeter length, which defines an important component of fire‐related edge habitat.

Fire severity is principally influenced by weather, but also by topography and fuel load (Bradstock et al., 2010). Fire extent and severity interact to generate a particular configuration of burnt, partially‐burnt and unburnt areas, collectively referred to as patchiness. Internal patchiness will influence the spatial pattern (size, shape, and contrast) of intra‐fire edges, but patchiness can also be conceptualized at a landscape scale as different fires burn and extinguish through time. Many fire management strategies aim to increase landscape variability by creating temporally and spatially dynamic fire mosaics often referred to as patch mosaic burning (Bradstock et al., 2005; Di Stefano et al., 2013; Parr & Andersen, 2006). However, the influence of patch mosaic burning on fire edges and how these might affect animal species and communities has not yet been considered as part of this paradigm.

Fire frequency (related to time‐since‐fire and inter‐fire interval) influences the temporal and spatial flux of fire edges. Fire frequency can affect the physical properties of edges (e.g., contrast) due to its interaction with climate, particularly post‐fire rainfall which contributes to edge regeneration.

Seasonality of fire influences edge architecture due to its effect on fire severity and patchiness, as well as the rate at which plants regenerate post‐fire. Unplanned fires more commonly occur in the driest months due to the seasonal growth and curing of fuel. Ease of ignition and flame transfer are also increased by high temperatures and low humidity common during summer (Bradstock, 2010). However, seasonality can also be affected by prescribed burning activities which often occur in different seasons to wildfires. Prescribed burning is usually undertaken early or late in the dry season when weather conditions are generally milder, more stable, with adequate fuel moisture to result in low‐intensity fire (Penman et al., 2011).

### 5.3 Edge architecture

The architecture of an edge refers to the physical characteristics of an edge zone, including size (volume) or depth‐of‐influence, shape, and contrast. Architecturally different edges are predicted to have equally divergent edge effects. Edges resulting from fire are likely to be compositionally diverse due to inherent variability in fire behavior in different landscapes and under different climatic conditions.

The volume of an edge (length, width, and height) is expected to affect the willingness of animals to cross it. Edge volume may alter foraging success and exposure to predation at small spatial scales, and metapopulation dynamics at large spatial scales (Nams, 2011).

Edge shape will likely influence permeability, with tortuous edges being more permeable than straight ones (Fagan, Fortin, & Soykan, 2003). For example, meadow voles (*Microtus pennsylvanicus*) crossed concave edges twice as often as straight or convex edges (Nams, 2012). Edge shape can also either concentrate or disperse animals, depending on species‐specific edge responses. In modified systems, species that are attracted to edges are more likely to collect in convexities and disperse from concavities, while the opposite is largely true for animals that avoid edges (Nams, 2011).

Edge contrast refers to the differences in structure and composition between adjoining parts of the landscape (i.e., between different vegetation growth stages) and is a key element influencing the movement of material, energy, and organisms (Villaseñor, Driscoll, Escobar, Gibbons, & Lindenmayer, 2014). In flammable landscapes, edge contrast is strongly influenced by topography, climate, and fire severity. Fire edges are likely to have high contrast immediately postfire (Figure 1c), and this contrast is expected to decrease over time as burnt parts of the landscape regenerate. However, different vegetation types have different regenerating capacities (i.e., resprouters compared to seeders) and differing levels of resilience to fire (i.e., forests are likely to support more vegetation structure post‐fire than grasslands). However, animals themselves can also influence edge contrast. Many grazing animals are attracted to recently burnt areas (Meers & Adams, 2003; Savadogo, Sawadogo, & Tiveau, 2007) and intense post‐fire grazing can help to maintain the divergence in structure between burnt and unburnt areas. Topography can also play a role in determining edge contrast. For example, in the temperate regions of the southern hemisphere edges on north‐facing slopes are likely to be characterized by stronger edge contrast than those on south‐facing slopes due to increased radiation and lower moisture, resulting in increased flammability.

### 5.4 Species traits

Species edge responses are usually attributed to physical architecture and biotic dynamics, but are also likely to be influenced by a species ability to perceive boundaries (Baguette & Van Dyck, 2007), as well as a series of morphological, behavioral and life‐history traits. How species respond to fire edges will be a function of their mobility (Ries et al., 2004), but also habitat and diet requirements, adaptability to disturbance, and susceptibility to other processes such as competition and predation.

Highly mobile organisms are more likely to survive edge creation compared to sessile species. Mobility and body size have an allometric relationship with metabolic rate, energy use, and physical ability, with larger animals generally requiring bigger home ranges than smaller animals (Lehman, Rajaonson, & Day, 2006). Larger body size might require foraging over large areas, increasing the chances of encountering more edge habitat, however, larger animals may be more able to cross edges and exploit adjacent habitat than smaller ones (Lees & Peres, 2009).

Diet specificity and habitat associations will also influence species edge responses. Species with more specialized requirements have been shown to avoid fire‐affected areas until important resources re‐accumulate (Borchert & Borchert, 2013). In contrast, species with generalist food or habitat requirements are predicted to fare better in a newly disturbed environment due to their ability to exploit a broader range of resources.

Fire edges may also influence the thermal landscape, potentially exacerbating fire edge effects for some species. In fragmented landscapes, patch edges frequently experience higher average temperatures and larger thermal variability than that of patch interiors (Tuff, Tuff, & Davies, 2016). Reduced vegetation cover after fire may cause species with narrow temperature thresholds to avoid the burnt side of edges for the first few years after fire. However, some species (such as ectotherms) may benefit from the contrast occurring at fire edges by “shuttling” (Dreisig, 1984) across burnt and unburnt edges to regulate body temperature. Understanding species thermal sensitivities and temperature thresholds will improve our ability to predict species responses to edges created by fire.

### 5.5 Edge dynamics

#### 5.5.1 Permeability/connectivity

Edge permeability ‐the ease with which animals, materials, and energy cross a boundary‐ has a key influence on species movement (Nams, 2012). Edges are often characterized by the rate at which they facilitate or impede movement of resources and organisms, processes that are strongly influenced by edge architecture. A hard edge is a boundary that individuals may find difficult to cross, whereas a soft edge will be reasonably permeable to most animals. The degree to which materials, energy, or organisms can flow across an edge has been largely attributed to vegetation structure (Cadenasso, Pickett, Weathers, & Jones, 2003); however, characteristics of the animals themselves (e.g., resource requirements or physical traits) can also influence boundary permeability, predominantly through changing rates of predation and competition.

Edge permeability is a site‐specific concept and its expression in time and space influences connectivity, determining the capacity of populations to move across landscapes. Edge permeability and landscape connectivity are the results of interactions between biophysical properties, components of a disturbance regime and the physical architecture of edges. Landscape connectivity is thought to depend on how an organism perceives and responds to landscape structures at various spatial scales (Bélisle, 2005). The ability of animals to cross fire edges, access available refuges and recolonize burnt landscapes will be influenced by both small‐scale permeability and the functional connectivity of the wider landscape. Unburnt refuges may sustain source populations that can recolonize burnt landscapes (Robinson et al., 2013); however, recolonization rates may be influenced by the permeability of an edge for the species in question.

#### 5.5.2 Ecological flows, access to resources and species interactions

The key dynamics commonly affected by edges are ecological flows, access to resources and species interactions. Edges can amplify, diminish or reflect ecological flows (Ries et al., 2004) and the rate at which these dynamics are affected is largely a function of edge permeability. Changes in processes occurring at edges can result in increased or reduced access to resources for some species, thereby changing the nature of species interactions. Changes to resource availability at edges can influence interspecific competition and alter community composition (Youngentob, Yoon, Coggan, & Lindenmayer, 2012). For example, predators are known to exploit recently burnt areas (Hradsky et al., 2017; McGregor, Legge, Jones, & Johnson, 2014), as reduced cover increases access to structurally complex habitats and therefore better hunting opportunities (Doherty, Davis, & van Etten, 2015). Edges are known to increase predation risk for many species, particularly for birds (Fisher & Wiebe, 2006; Vetter, Rücker, & Storch, 2013), and small mammals (Hof, Snellenberg, & Bright, 2012; Kingston & Morris, 2000; Šálek, Kreisinger, Sedláček, & Albrecht, 2010). Accordingly, animals living near edges may alter their behavior to compensate for high predation risk, such as decreasing their use of high‐exposure locations or reducing visually conspicuous behaviors (Anderson & Boutin, 2002).

### 5.6 Species edge response

The culmination of all the factors listed above results in a species’ edge response, which is commonly described as being positive, neutral or negative (Ries et al., 2004). Edge response can be considered at the community, species or individual level. Multi‐species edge responses are often reported using a measure of community composition such as species richness. Single species responses are usually measured as a change in occupancy, abundance or behavior, and may be partitioned further into sex or age‐specific effects. Both single and multi‐species edge responses occur along a continuum of spatial and temporal scales.

## 6 FUTURE RESEARCH

Our model provides a conceptual understanding of edge creation in flammable landscapes and associated implications for fauna. However, few data quantifying these processes exist, providing several avenues for future research.

### 6.1 Understanding fire as an agent of edge creation

The agent of edge creation can strongly influence ecological patterns and processes, but few edge‐related studies have considered how the process of creation influences edge effects. Understanding how fires interact with biophysical properties to create edges is an important first step in future fire edge research.

### 6.2 Modeling the spatio‐temporal flux of fire edges in flammable landscapes

Edges in highly modified systems are often maintained at a relatively stable state, whereas edges in natural systems are dynamic, changing both spatially and temporally. In flammable landscapes, modeling the effect of fire cycles and plant regeneration rates on the distribution, abundance, and architecture of edges will be an important precursor to understanding fire‐induced edge effects more broadly, particularly at landscape scales.

Fire edges need to be studied at multiple spatial and temporal scales. Understanding how permeability at single edges interact to influence landscape‐scale structural and functional connectivity will be important for the conservation of biodiversity in fire‐prone systems, particularly when considering landscapes that contain complex and varied fire histories.

### 6.3 Understanding the effect of fire edges on edge dynamics

Ecological flows, resource selection, and species interactions are predicted to be influenced by edge architecture. However, the interaction between edge architecture and edge dynamics has not yet been studied in flammable ecosystems. Better knowledge of these relationships will aid our understanding of the underlying mechanisms that drive species and community edge responses in fire‐prone landscapes.

## 7 CONCEPTUAL MODELS AS BAYESIAN NETWORKS: ENHANCING FUTURE RESEARCH

To direct future research efforts our conceptual model has been designed as a Bayesian Network (BN). A BN is a statistical framework capable of analyzing complex environmental relationships between a range of variables (Penman, Price, & Bradstock, 2012). BNs have strong predictive power where empirical datasets are incomplete and can be used to predict species responses in cases where potential drivers are correlated. In the absence of a complete dataset, a BN will allow the sensitivity of the output to be tested against different factors by inserting a range of possible values into each node. This will identify the most influential nodes, generating hypotheses and highlighting focal points for further research. Further, the conceptual model can become a numerical model as empirical data are acquired, strengthening predictive outputs over time.

In our conceptual model, we propose that biophysical properties interact with the fire regime to influence edge architecture. Edge architecture and species traits influence edge permeability, which affect important processes (i.e., movement, resource selection and species interactions) that influence both species and community‐level edge responses. Conversion of the conceptual model into a BN will enable these conceptual advances to be effectively combined with new data as fire edge research is conducted. The capacity of the BN to deal with data gaps and to be used as a hypothesis generation tool will be particularly useful given the current paucity of information about faunal responses to fire edges.

## CONFLICT OF INTEREST

None declared.

## AUTHOR CONTRIBUTIONS

K.P, J.D, and A.Y conceived the ideas and developed the structure. K.P led the writing of the manuscript. All authors contributed critically to the drafts and gave final approval for publication.
